# Effect of a Text Messaging–Based Educational Intervention on Cesarean Section Rates Among Pregnant Women in China: Quasirandomized Controlled Trial

**DOI:** 10.2196/19953

**Published:** 2020-11-03

**Authors:** Yanfang Su, Jesse Heitner, Changzheng Yuan, Yafei Si, Dan Wang, Zhiying Zhou, Zhongliang Zhou

**Affiliations:** 1 School of Medicine University of Washington Seattle, WA United States; 2 Aceso Global Washington, DC United States; 3 The Children’s Hospital and School of Public Health Zhejiang University School of Medicine Hangzhou China; 4 Nutrition Department Harvard School of Public Health Boston, MA United States; 5 School of Risk & Actuarial Studies and Centre of Excellence in Population Ageing Research (CEPAR) University of New South Wales Sydney Australia; 6 School of Public Policy and Administration Xi’an Jiaotong University Xi'an China; 7 School of Public Health Xi’an Jiaotong University Health Science Center Xi'an China

**Keywords:** cesarean section, short message service, SMS text messaging, quasirandomized controlled trial, mobile health

## Abstract

**Background:**

Consensus exists that appropriate regional cesarean rates should not exceed 15% of births, but China’s cesarean rate exceeds 50% in some areas, prompting numerous calls for its reduction. At present, China’s 2016 two-child policy has heightened the implications of national cesarean section trends.

**Objective:**

This study leveraged pervasive cellular phone access amongst Chinese citizens to test the effect of a low-cost and scalable prenatal advice program on cesarean section rates.

**Methods:**

Participants were pregnant women presenting for antenatal care at a clinic in Xi’an, China. Assignment was quasirandomized and utilized factorial assignment based on the expecting mother’s birthday. Participants were assigned to one of the following four groups, with each receiving a different set of messages: (1) a comparison group that received only a few “basic” messages, (2) a group receiving messages primarily regarding care seeking, (3) a group receiving messages primarily regarding good home prenatal practices, and (4) a group receiving text messages of all groups. Messages were delivered throughout pregnancy and were tailored to each woman’s gestational week. The main outcome was the rates of cesarean delivery reported in the intervention arms. Data analysts were blinded to treatment assignment.

**Results:**

In total, 2115 women completed the trial and corresponding follow-up surveys. In the unadjusted analysis, the group receiving all texts was associated with an odds ratio of 0.77 (*P*=.06), though neither the care seeking nor good home prenatal practice set yielded a relevant impact. Adjusting for potentially confounding covariates showed that the group with all texts sent together was associated with an odds ratio of 0.67 (*P*=.01). Notably, previous cesarean section evoked an odds ratio of 11.78 (*P*<.001), highlighting that having a cesarean section predicts future cesarean section in a subsequent pregnancy.

**Conclusions:**

Sending pregnant women in rural China short informational messages with integrated advice regarding both care-seeking and good home prenatal practices appears to reduce women’s likelihood of undergoing cesarean section. Reducing clear medical indications for cesarean section seems to be the strongest potential pathway of the effect. Cesarean section based on only maternal request did not seem to occur regularly in our study population. Preventing unnecessary cesarean section at present may have a long-term impact on future cesarean section rates.

**Trial Registration:**

ClinicalTrials.gov NCT02037087; https://clinicaltrials.gov/ct2/show/NCT02037087.

**International Registered Report Identifier (IRRID):**

RR2-10.1136/bmjopen-2015-011016

## Introduction

The global health care community has estimated that regional cesarean section (CS) rates should not exceed 10% to 15% [1,2]. However, in the Global Survey by the World Health Organization (WHO), CS in China was estimated to involve 46.2% of all deliveries, which is the highest rate for any country in the survey [3]. In a recent multicenter survey of 39 hospitals across mainland China, the overall CS rate was 54.90% [4]. It is estimated that between 1990 and 2014, China had an average annual rate of increase in CS of about 10%, and misconceptions of pain, genital modification, safety, and cultural fortune were reported as leading factors [5]. Since at least 2008, this increase was most visible in the rural counties of China [6]. Though rural counties have clearly lower rates of CS than general city or “supercity” areas, as of 2014, the average rural county CS rate was slightly over 30%, and it is rising [6].

When warranted, CS is a vital intervention. Many studies have confirmed that it has a strong protective effect on perinatal mortality when conditions, such as breech presentation, placenta previa, and uterine rupture, are encountered [3,7,8]. A study of 66,226 deliveries in Shanghai found that compared with vaginal delivery, CS was associated with a reduction in antepartum stillbirth, bone trauma, intracranial hemorrhage, and neonatal hypoxemic encephalopathy [9]. Moreover, studies suggest no evidence of a difference in maternal mortality between *planned* vaginal and *planned* cesarean delivery [10]. In the Global Survey by the WHO, the maternal mortality risk for antepartum CS without an indication could not be estimated because there were no maternal deaths in the group [3]. However, CS does come with serious risks [8]. Using data from the Global Survey by the WHO, although researchers found no association with maternal *mortality*, CS without medical indications had strong associations with severe maternal *morbidity*. On putting death and several severe morbidities, namely admission to the intensive care unit, blood transfusion, and hysterectomy, into one “Severe Maternal Outcomes” index, the authors found that elective antepartum CS had an adjusted odds ratio (OR) of 5.93 (CI 3.88-9.05) for qualifying for the index and elective intrapartum CS had an adjusted OR of 14.29 (CI 10.91-18.72) (both *P*<.05) [11]. A large cohort study in Australia found that mothers delivering via CS were more likely to be readmitted to the hospital within 8 weeks of birth [12].

CS is also associated with problems after delivery. A recent study found that women delivering via CS had roughly twice the odds of persistent pain 1 year after delivery [13]. The association between CS and reduced future fertility has been demonstrated in numerous studies [14]. In a new pregnancy, a *prior* CS may cause an increased risk of fetal wastage and may be linked to unexplained stillbirth [10,14]. Further, there is a strong body of evidence on impaired uterine function following cesarean delivery. In subsequent pregnancies, CS poses a risk of uterine scar dehiscence, and, in some cases, uterine rupture [14]. As of January 2016, China altered its one-child policy to a two-child policy, suddenly making the effects of CS on subsequent pregnancies a tremendously more important consideration.

Given the risks and benefits, the WHO has concluded that “cesarean section should ideally only be undertaken when medically necessary” [2]*.* Yet, Lumbiganon et al estimated that 11.7% of all deliveries in China during their study period involved CS with no medical indications [3]. Combining the 24 countries in the Global Survey by the WHO, it was estimated that 63% of all CS procedures without medical indications were performed in China [11]. An important driver of this pattern is that women with no indications necessitating CS frequently request this procedure. Cesarean delivery on maternal request accounted for 15.53% of all deliveries and 28.43% of all cesarean deliveries in a multicenter survey [4]. This national estimate confirms what at least 11 other smaller and qualitative studies [15] and at least one regional estimate [16] have suggested (women’s preferential choices are part of the rise in China’s CS rates). Influencing this demand may be an important strategy for reducing medically unnecessary CS.

There is also evidence that part of the “demand” for CS is supplier induced [17]. CS brings in approximately double the hospital revenue per birth as compared with vaginal delivery [16,18], and the power imbalance between patients and providers may mask the true decision making [14,15]. In a recent study in Shanghai, of 599 women interviewed in their third trimester, 17.0% reported preferring cesarean delivery. Yet, among women completing the study, 58.1% underwent CS. Of those, only 50.0% had clinically accepted indications for CS [19]. Therefore, educating and empowering women to refute inappropriate doctor recommendations for CS may be as important of a pathway for reducing CS as changing women’s underlying preferences.

There is agreement that the rate of CS in China, whether supply or demand-side driven, is excessive, and experts are calling for strategies for its reduction [1,4,17]*.* Mobile health (mHealth) generally has already shown relevant effects in several intervention areas. However, evidence for or against its efficacy for maternal and child health is scarce [20,21], and larger scale evaluations of its possible effects are warranted [22-27]. This study comprises a portion of the Evaluation for mHealth Interventions’ Newborn Health Project. The Newborn Health Project has several aims, and its primary (trial registered) metric of success is a newborn’s appropriate weight for gestational age [28,29]. This study investigated whether the project was successful in the secondary goal of lowering the rate of CS in the intervention arms.

## Methods

The Newborn Health Project offers expectant mothers in the rural district of Gaoling in Xi’an, China, a package of free, short, informational messages regarding pregnancy and childbirth via cell phones. The full protocol of the Newborn Health Project has been published elsewhere [29], including the process and rationale for selecting the study site. The study utilizes factorial quasirandomization at the individual level to assign women to receive one of four groups of text messages and then compares outcomes between the four groups. Participating women were blinded to assignment. All data analyses were performed with blinding to treatment assignment. The trial has been registered at ClinicalTrials.gov (NCT02037087).

The four study arms were as follows: (1) good home prenatal practice messages (home practices), which included advice on nutrition, exercise, self-awareness of depression, breastfeeding, etc; (2) care-seeking messages (care seeking), which included information about government-subsidized programs, warning signs of potential problems, and the importance of care seeking during illness; (3) both types of messaging (all texts); and (4) a very limited (25 in total) set of “basic” messages about pregnancy (acting as a comparison group). Women in the other intervention arms also received all of the basic messages. It was decided that the comparison group should receive at least some regular informational “placebo” messages to make the participants in this group feel like they received a service and were part of the program. Ethically, it also ensured that all enrollees received the most basic pregnancy information, which is the informational equivalent of “basic care.” These basic messages primarily included updates on fetal development, as well as reminders for prenatal visits and promotion of certified skilled attendance of labor. Group comparisons of treatment arms elicited the effect of (assignment to) receiving the *content* in the intervention messages in addition to the basic ones, and estimated this effect separated out from any effect associated with being included in an informational messaging study. The number of messages by topic and study arm is presented in Multimedia Appendix 1.

The four treatment arms received differing sets of messages relevant to labor and delivery that could potentially impact a woman’s choice of delivery mode. Of the “basic” (comparison) group’s 25 messages, none were relevant to delivery. The “care-seeking” group was sent seven relevant messages, generally focusing on describing proper indications for CS and cautioning that CS and anesthesia make birth less painful but come with their other risks. The “home practices” group was sent 15 delivery-relevant messages, generally focusing on inspiring confidence in vaginal delivery and discussing nonanesthetic ways to cope with pain during delivery. The “all texts” group received 22 relevant messages composed of those sent to the other treatment arms. The sent messages relevant to labor and delivery are presented in Multimedia Appendix 2. Though presented in English there, all sent messages were in Mandarin.

This study was approved by the Ethics Committee of the School of Medicine at Xi’an Jiaotong University on January 18, 2013, and an updated version was approved on May 2016 (approval number: 2016-392). All women attending their first visit for antenatal care at Gaoling district’s local maternal and child health center (MCHC) during the study period were invited to participate if they were aged 18 to 45 years old and had access to a cellular phone within their household. All participants signed an informed consent form. Pilot testing with 140 subjects occurred between September and October 2013, and survey questionnaires were finalized after incorporating feedback from the testing. The participants included in our pilot were excluded in our data analysis and causal inferences. Enrollment and data collection for the study were performed between November 2013 and February 2016. Enrollment stopped before our power calculation–based sample size goals were reached in December 2015, when Gaoling MCHC stopped sending program texts after decided that future patient communications should preferably be sent over WeChat than cellular SMS text messaging. It is extremely noteworthy that the contents of our SMS intervention could easily be sent via WeChat, but in December 2015, the trial was not set up to do so.

At recruitment, prior to treatment assignment, a baseline survey was conducted by a health worker at the local MCHC during the first antenatal visit. We collected demographic data, self-reported health data, and data relating to each enrollee’s thoughts and perceptions regarding health during pregnancy and childbirth. The follow-up survey was conducted by a health worker at the newborn’s home around 1 week after delivery. The follow-up survey collected information on knowledge, psychological and behavioral changes, and pregnancy-related maternal and neonatal measures. A final survey was conducted by phone to assess postpartum depression around 1 month after delivery. Additionally, the final survey asked whether the enrollee had successfully received our messages.

## Results

**Figure 1:**
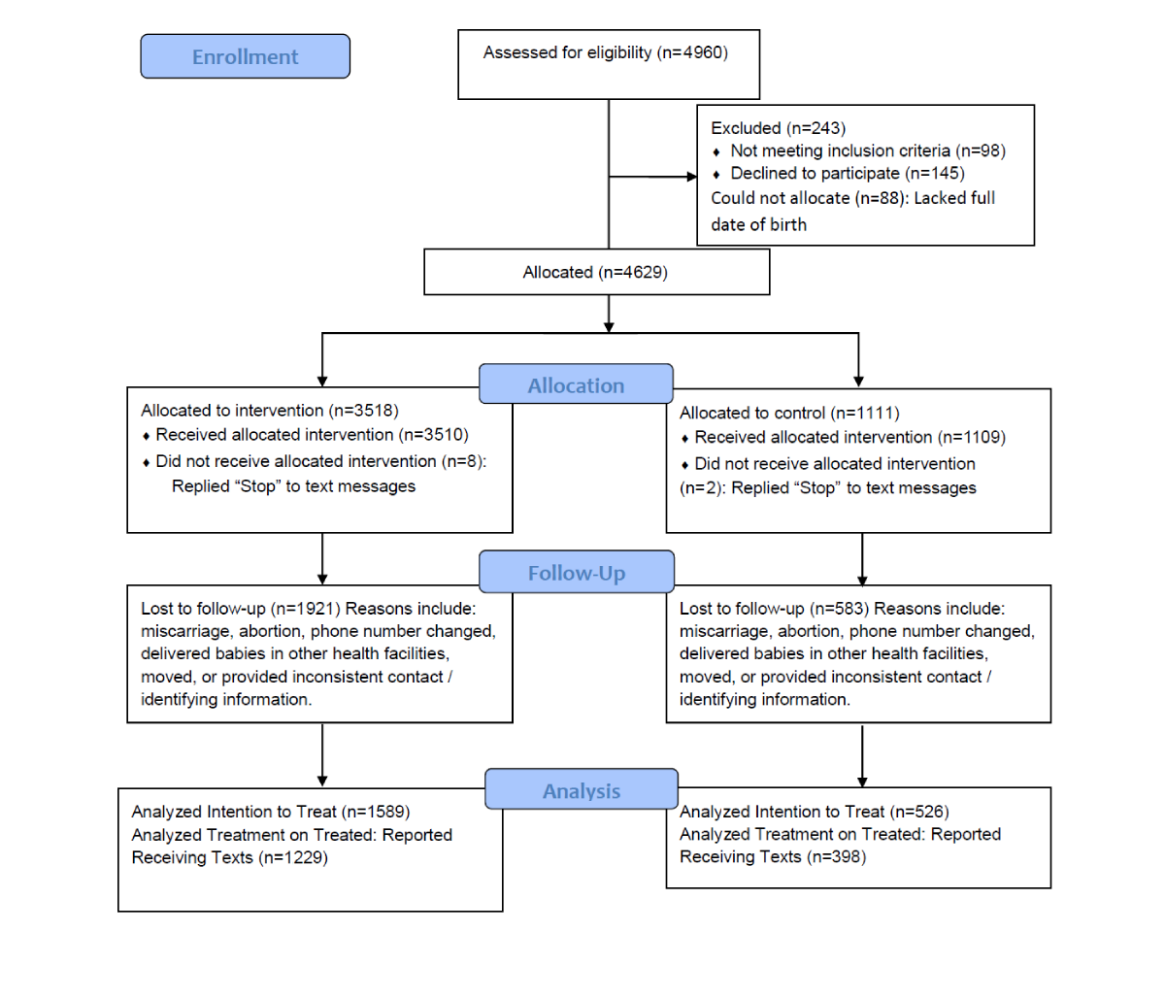
CONSORT flow diagram: enrollment, allocation, follow-up, and analysis.

Our program enrolled a total of 4629 women (Figure 1). In our prior publication and Multimedia Appendix 3 [28], we present summary statistics and balance checks for all 55 measured baseline covariates. As previously reported via these balance checks, we inferred that our quasirandomization was effective in assigning treatment orthogonally to relevant observable covariates.

In total, 2115 women completed a postdelivery follow-up survey, which could be linked to the baseline survey (Figure 1). Among these, 526 (24.9%) were in the “basic” group, 518 (24.5%) in the care-seeking group, 497 (23.5%) in the home practices group, and 574 (27.1%) in the all texts group. A chi-square test failed to reject equal loss to follow-up at *P*=.44. A balance check on all measured baseline characteristics was performed restricted to the final 2115 women. Only one test rejected balance at *P*<.05, and a further three rejected balance at .05<*P*<.10. We therefore inferred that among the women completing our study, our quasirandomization was effective in assigning treatment orthogonally to observable covariates. This set of balance checks is presented in Multimedia Appendix 4.

Loss to follow-up occurred via multiple pathways. First, only women who delivered their children, including stillbirths, were available for analysis regarding mode of delivery. This analysis was inapplicable for any woman who miscarried or underwent an abortion after enrollment. About 15% of clinically recognizable pregnancies end in spontaneous miscarriage within the first trimester [30]. As of 2010, China’s induced abortion rate was hovering steadily around 19.5% [31]. Rates of miscarriage and abortion were not collected as part of our trial, but likely account for important shares of our loss to follow-up. Further, if women moved or otherwise gave birth outside of our study catchment area or if the village health attendant was otherwise unaware of a birth or unable to reach a new mother at her home, the pregnant woman was lost to follow-up. Finally, it was sometimes impossible to match follow-up surveys to baseline surveys. This happened when women provided sufficiently different identifying information in the baseline and follow-up surveys or when such information was sufficiently misrecorded.

The unadjusted rates of cesarean delivery by treatment assignment are presented in Table 1. The largest difference was between the “basic” group and the “all texts” group, whose rate was lower by 5.2 percentage points.

**Table 1:** Cesarean section numbers, rates, and odds ratios by treatment assignment, among women who reported delivery mode.

Group	Women who completed follow-up survey, n	Women who reported delivery mode, n	Vaginal deliveries, n	Cesarean sections, n (%)	Cesarean, odds ratio
Basic	526	522	372	150 (28.7)	Base case
Care Seeking	518	515	379	136 (26.4)	0.88
Home Practices	497	494	>369	>125 (25.3)	0.84
All Texts	574	>570	>436	>134 (23.5)	>0.77
Total	2115	2101	1556	545 (25.9)	N/A^a^

^a^N/A: not applicable.

Only 2101 of 2115 women reported their mode of delivery (Figure 1). Some missingness is to be expected in any large-scale survey, and no variable measured had all 2115 responses. It has been shown that a process called “multiple imputation” using expectation maximization will generally outperform the most common general techniques of handling missing data [32]. Thus, multiple imputation was performed in R (R Foundation for Statistical Computing) using the Amelia package. This process imputed 16 data sets that had “complete” data on all variables of interest, allowing all 2115 observations to be used in regression analysis. All regression analyses were run once per imputed data set, and the results were combined using the Rubin technique for combining quantities of interest [32].

Four regression models were run to explore the impact of treatment on CS rates. Model 1 is an unadjusted logistic regression of the (log) odds of having CS on indicators for assignment to each intervention arm, with the “basic” arm omitted as the base case. Results are presented in Table 2.

In Model 1, neither the “care-seeking” group nor the “home practices” group alone was associated with a statistically significant reduction in the odds of undergoing CS (*P*=.37 and *P*=.21, respectively). In combination, the all texts group was associated with an OR of 0.77, but the *P* value was .06.

A second model, also presented in Table 2, included all baseline covariates found to be unbalanced at *P*<.10 for either the full sample or for the 2115 subjects who completed the study. Adding these unbalanced covariates had negligible effect on regression results. The *P* value on assignment to the all texts group reached .048, but accounting for multiple comparisons left this finding not statistically significant. One noteworthy finding is that having a previous miscarriage was strongly predictive of undergoing CS at our study site, with an associated OR of 1.37 (95% CI 1.12-1.68). Whether the mechanism of this association is physiological or psychological may be an area for future study.

As a further robustness check, a third logistic regression model (Model 3) was run, which contained all unbalanced baseline covariates as well as an array of baseline general health, maternal, socioeconomic, and health psychology covariates that might influence or predict birth via CS. These results are also presented in Table 2. The health psychology covariates (not shown) attempted to account for the major constructs of the most widely cited theories of health behavior, namely the health belief model, social cognitive theory, theory of planned behavior, theory of reasoned action, and trans-theoretical model [33-35].

**Table 2:** Logistic regression analysis of cesarean birth according to treatment assignment.

Model					Odds ratio			95% CI			*P* value	
**Model 1: Unadjusted logistic regression**												
	**Treatment assignment**											
		Basic only	Base case			N/A^a^			N/A			
		Care seeking	0.88			0.67-1.16			.37			
		Good home practices	0.84			0.63-1.11			.21			
		All texts	0.77			0.59-1.01			.06			
**Model 2^b^: Adjusted logistic regression for imbalance**												
	**Treatment assignment**											
		Basic only	Base case			N/A			N/A			
		Care seeking	0.87			0.66-1.14			.32			
		Good home practices	0.82			0.62-1.08			.16			
		All texts	0.76			0.58-1.00			.048			
**Model 3^c^: Adjusted logistic regression for all**												
	**Treatment assignment**											
		Basic only	Base case			N/A			N/A			
		Care seeking	0.75			0.54-1.04			.08			
		Good home practices	0.77			0.56-1.07			.12			
		All texts	0.67			0.49-0.92			.01			

^a^N/A: not applicable.

^b^Model 2 adjusted for all unbalanced baseline covariates.

^c^Model 3 adjusted for all unbalanced baseline covariates as well as an array of baseline general health, maternal, socioeconomic, and health psychology covariates (Multimedia Appendix 4).

These additional covariates noticeably strengthened the measured effect of assignment to each treatment arm. The OR for the care-seeking group fell from 0.87 to 0.75, the OR for the home practice group fell from 0.82 to 0.77, and the OR for the all texts group fell from 0.76 to 0.67. The *P* values on assignment to the first two groups remained above traditional significance (*P*=.08 and *P*=.12 respectively), but that of assignment to the all text group dropped to *P*=.01, which remained statistically significant under the Bonferroni method of correcting for multiple testing of three tests in the same regression.

Most of the magnitude of these changes can be accounted for by the addition of two variable sets (analysis not shown), including whether the woman previously had CS (OR 11.8, 95% CI 7.2-19.3) and indicators for her stated preferred mode of delivery during enrollment. Preferring CS over vaginal birth and feeling unsure are both associated with about twice the odds of CS (OR 2.0, 95% CI 1.3-3.2 and OR 2.0, 95% CI 1.4-2.9, respectively). Intriguingly, this equality implies that feeling “unsure” carries the same effect as actually preferring CS, perhaps implicating a strong supply-side nudge toward CS for uncertain women. An enormously pressing implication of Model 3 is that if a woman has CS in one pregnancy, she is vastly more likely to have one in any subsequent pregnancy, raising the possibility that preventing unnecessary CS now may have a direct and long-term impact on future CS rates.

As mentioned, the postdelivery survey also inquired whether the enrollees had actually received text messages from the Newborn Health Project during pregnancy. Surprisingly, of the 2115 participants, only 1627 (76.93%) answered “Yes,” 459 (21.70%) reported not receiving messages from the study, and 29 (1.37%) did not respond to that question. A final model was run with the same unadjusted functional form as Model 1, but limited to participants who answered “Yes” or were imputed to have answered “Yes.” The results are displayed in Table 3.

It is unknown why 459 of the 2115 participants (21.70%) reported not receiving messages from the study. Nonexclusive possibilities include phone numbers being miswritten on the survey, phone numbers being misentered into the SMS delivery system, participant phone numbers changing after enrollment, participants giving numbers beside their text-enabled cellular numbers as requested, and recall error.

This subset that reported receiving this study’s texts displayed a stronger unadjusted intervention effect than that for all study participants. In this subset (Figure 1), the all texts group was associated with a highly significant reduction in the odds of undergoing CS (OR 0.66, *P*=.008).

**Table 3:** Logistic regression analysis of cesarean birth according to treatment assignment (women who reported text receipt).

Treatment assignment (unadjusted logistic regression)	Odds ratio	95% CI	*P* value
Basic only	Base case	N/A^a^	N/A
Care seeking	0.76	0.55-1.04	.09
Good home practices	0.80	0.58-1.09	.12
All texts	0.66	0.49-0.90	.008

^a^N/A: not applicable.

## Discussion

### External Validity

After adjusting for potentially confounding covariates, our main finding shows that the group with all texts sent together had an OR of 0.67 (*P*=.01). The rate of CS in the basic (comparison) group of our study (28.7% between 2013 and 2016) is identical to that in a study in rural Shaanxi, China in 2018 [36] and is well representative of (slightly lower than) the typical rural county rate of just over 30% in China in 2014, which was reported by Li et al [6]. Nonetheless, it is roughly double the WHO’s recommended rate of 10% to 15%, and merits improvement. One possible reason for this disparity is that our sample came from an MCHC in a rural area. It has been estimated that women in large cities in China have 2.4 times the odds of CS as compared with women in smaller cities [37]. Further, Liu et al found that CS rates increased with hospital complexity, with tertiary care facilities having the highest rates [4]. Thus, our findings are likely to be most closely replicated in rural areas of China, but may be well indicative of such counties.

### Potential Pathways

**Figure 2:**
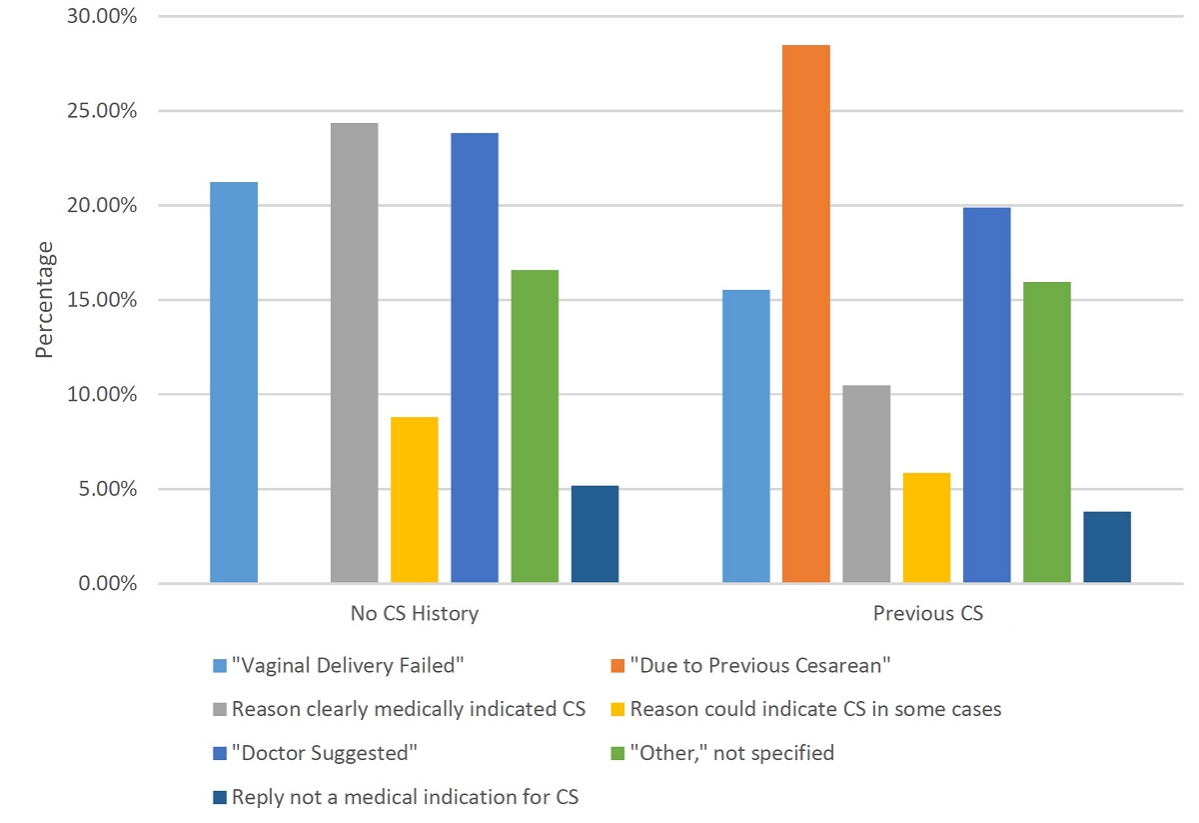
Reasons for current cesarean section (CS) delivery by CS history.

Our informational texts had several hypothesized pathways by which they might impact mode of delivery, including (1) altering women’s underlying preferences, (2) empowering women to decline doctor-suggested CS by increasing their knowledge and self-efficacy, and/or (3) reducing the number of legitimate indications for CS. Our follow-up survey asked women who delivered via CS why they did so, and investigating their responses shed some light on how our intervention may have been effective. Due to the very strong association between a reported previous CS and mode of delivery, we first present responses broken down by CS history in Figure 2.

There was an unadjusted increase in the probability of delivering via CS of 54 percentage points, from about 27% probability among women without a previous CS to about 81% probability given a previous CS. A previous CS is often considered its own indication for CS and accounted for the largest difference between the two groups, but women with a history of CS had more CS procedures for every single reason category. The percentages in Figure 2 were derived from compiling the imputed “complete” data sets. The raw unimputed data had a smaller sample but indicated an even more stark difference in group rates of CS. Because of this strong association, we hypothesize that CS history could act as an effect modifier for our SMS intervention, but we were not powered to test this hypothesis directly. Therefore, we present in Multimedia Appendix 5 the stated reasons for CS by intervention group only for those women with no stated history of CS, accounting for about 87% of our sample. For convenience, Multimedia Appendix 6 presents the same data in terms of each arm’s differences from the “basic” texts group.

CS based only on maternal request was not a common occurrence in our sample (Figure 2). Stated reasons for CS that were not a medical indication accounted for less than 5% of CS cases (26/548 cases) in our sample. This left our intervention very little room to affect CS by reducing cesarean delivery on maternal request, though the “other/not specified” category may or may not be comprised largely of maternal requests. Multimedia Appendix 6 shows that reducing clear medical indications for CS seems to be the strongest potential pathway for our texts to have had an effect, with this category showing the largest reduction in all treatment groups. The provider-induced “doctor-suggested” category had mixed evidence, being reduced in the “all texts” group but ostensibly increased in the individual “care-seeking” or “home practices” group.

### Limitations

Our study has several limitations. First, women self-reported their delivery method to health workers. If our intervention induced a belief that vaginal delivery was a more socially desirable answer than CS, CS could be underreported in intervention arms. Second, our study population was concentrated in a single county. It is unknown how results would differ in other settings. Finally, high loss to follow-up lowered the planned statistical power of our study. Attrition does not seem to be associated with treatment assignment, and baseline covariates were balanced within both the full sample and the subset that completed the study, which suggests that loss to follow-up did not bias our results. However, we cannot measure whether it is associated with the mode of delivery, and as such, we cannot confidently rule out the possibility that high attrition altered our findings.

### Implications

As a first of its kind, this evaluation breaks ground in the fields of SMS text messaging for maternal health in China and SMS text messaging for influencing the mode of delivery. In 2015, China had 16.55 million new births [38]. With the recent relaxations in China’s one-child policy, this number could grow considerably in the next few years. Given the risk that unnecessary CS poses and the current excessive amount in China, wider distribution of the Newborn Health Project’s messages on delivery mode seems to be a strategy worth trying. The acceptability and effectiveness of WeChat as a mode of delivering such messages also warrant scientific exploration.

### Conclusions

A quasirandomized controlled trial distributing informational text messages to pregnant women in Gaoling, China found evidence that the full set of text messages may have reduced the number of cesarean deliveries in that group by 5.2 percentage points compared with the comparison group. Focusing on the subset of women who reported actually receiving program texts and adjusting for baseline covariates greatly strengthened this measured relationship. Given the numerous calls for strategies to reduce the rate of medically unnecessary CS in China, exploration of the wider distribution of these text messages seems warranted.

## Appendix

Multimedia Appendix 1 SMS text messages by general topic, treatment group, and timing.

Multimedia Appendix 2 Messages regarding delivery advice by treatment arm.

Multimedia Appendix 3 Balance check and all baseline variables: all enrollees.

Multimedia Appendix 4 Balance check and all baseline variables: women with follow-up surveys.

Multimedia Appendix 5 Reasons for current cesarean section delivery by SMS intervention assignment (N=2104).

Multimedia Appendix 6 Differences in reasons for current cesarean section delivery by SMS intervention assignment.

Multimedia Appendix 7 CONSORT-EHEALTH checklist (V 1.6.1).

## Abbreviations

CS: cesarean section
MCHC: maternal and child health center
mHealth: mobile health
OR: odds ratio
WHO: World Health Organization
