# Supplementary material for: Effect of a Text Messaging–Based Educational Intervention on Cesarean Section Rates Among Pregnant Women in China: Quasirandomized Controlled Trial
Source: JMIR Mhealth Uhealth. 2020 Nov 3;8(11):e19953. doi: 10.2196/19953 (PMC7671841; doi:10.2196/19953)
Supplement: Multimedia Appendix 1 [file mhealth_v8i11e19953_app1.pdf]

Multimedia Appendix 1. SMS messages, by general topic, treatment group, and timing

Randomized group	Message categories	Message delivery time and total number of SMS messages				
		Sign-up day	First trimester	Second trimester	Third trimester	Final day
**Basic group (25)**	Fetal development (19)	2	6	6	3	2
	Reminders for prenatal visit and hospital delivery (6)		1	1	4	
**Care-seeking group (82)**	Fetal development (19)	2	6	6	3	2
	Reminders for prenatal visit and hospital delivery (8)		2	2	4	
	Warnings & Recognition of danger signs (45)		5	23	17	
	Reminders for government-subsidized projects (10)		3	2	5	
**Good household prenatal practice group (91)**	Fetal development (19)	2	6	6	3	2
	Reminders for prenatal visit and hospital delivery (6)		1	1	4	
	Healthy lifestyle (Nutrition, physical activity, etc.) (37)		15	16	6	
	Mental health during pregnancy (8)		1	4	3	
	Pain management (9)			4	5	
	Labor (6)			3	3	
	Breastfeeding (6)				6	
**All texts: full SMS bank (148)**	Full bank (148)	2	32	60	52	2
