# Supplementary material for: Effect of a Text Messaging–Based Educational Intervention on Cesarean Section Rates Among Pregnant Women in China: Quasirandomized Controlled Trial
Source: JMIR Mhealth Uhealth. 2020 Nov 3;8(11):e19953. doi: 10.2196/19953 (PMC7671841; doi:10.2196/19953)
Supplement: Multimedia Appendix 2 [file mhealth_v8i11e19953_app2.pdf]

Multimedia Appendix 2 Messages regarding delivery advice by treatment arm

Comparison group / basic
None

Care seeking
Day 158: Cord around the neck is a common, ultrasound, fetal movement monitoring can help diagnose it. If abnormal, please seek immediate medical attention, and choose the appropriate delivery method with the guidance of the doctor.

Day 205: Vaginal delivery is the most reliable delivery method unless doctor suggests you to use c-section. Although anesthesia in C-section would cause no pain during your delivery, you will still be painful during recovery. Usually, C-section takes more time to recovery. If you feel much pain after delivery, you could take painkilling medicines according to the doctor's prescription.

Day 207: Vaginal bleeding in the third trimester may indicate placenta previa, if diagnosed by ultrasound, you should take more rest, avoid fatigue and vaginal stimulation. If necessary, you may need caesarean section.

Day 209: Don't panic about baby malposition. Sometimes malposition can be self-corrected. As long as you go to regular prenatal care, and choose the right delivery approach according to your doctor, you can deliver safely.

Day 223: The use of anesthesia for "painless childbirth" can reduce pain, but it can also bring risks. Experts suggest less intervention and a more natural way of labor. Avoid using medicine or surgical operations unless there are medical reasons for doing so.

Day 263: Doctors will decide whether you should have early admission, fetal monitoring and your delivery approach by measuring the inside and outside of the pelvis and ultrasonography. If doctors suggest you to have vaginal delivery, please don't say no just because of fear of pain.

Day 268: If you have passed the expected due data, please do not worry. Only 5% of babies are born within the due date. The doctor will use ultrasound and fetal heart rate monitor to check the safety of pregnancy.

Home practices
Day 104: Breathing training can help you stay relaxed and calm during childbirth. Rama breathing exercise is the most commonly used, and it can help reduce the pain during labor. Key message: Take a deep nasal breath with your nose, and then exhale slowly through your mouth. Please consult the Maternal and Infant Healthcare Center for details.

Day 121: Prenatal classes on delivery are also a good way to help you understand the labor process. To be more prepared, you can take a prenatal class in addition to our 'Baby Letter'.

Day 140: Good control of hormone levels will help reduce the pain. During labor, please keep calm and confident, which could help regulate the secretion of hormones, thereby reducing pain.

Day 163: There are three main ways to deliver a baby: natural childbirth (without anesthesia); painless childbirth (using local anesthetic); caesarean section. If no high risk factors, it is better to choose natural childbirth. If you want to use natural birth but are very afraid of the pain, you can learn more about how to reduce pain during childbirth.

Day 172: For most normal pregnancy, vaginal delivery is the most reliable method, and is more beneficial for the mother's recovery compared to C-section, it also increases the success of breastfeeding.

Day 177: Pelvic exercises will make your childbirth easier and help prevent complications such as perineal tear. Pelvic exercises have the following steps: contraction of the anus and vagina, then relax. You can practice it whenever you have time.

Day 200: Vaginal delivery is the most reliable delivery method. For most of the normal pregnancy, natural childbirth is better for recovery, more beneficial for baby and has a higher rate of successful breastfeeding compared to caesarean section.

Day 202: Still remember Lamaze breathing exercises? Do not forget to keep training your breathing? Key points are to take a deep breath through nasal, then exhale through your mouth slowly. This can help stay relaxed and calm during childbirth, can reduce the pain from production.

Day 210: Do you have confidence in the delivery? Do you feel a little worried or panic? Tell your concerns to your doctor, and learn some information about labor and delivery in advance. You will feel more comfortable with someone familiar around you.

Day 224: When delivering a baby, in addition to using local anesthetic, there are many other ways to relieve pain, such as deep breathing, massage, and try different production poses.

Day 237: During labor, lower back massage can reduce pain. But also can massage the head and face, to relax and unwind.

Day 247: You don't have to lie on the bed before labor. You can walk, stood, swaying body in the delivery room, leaning against the wall leaning, kneeling, or lying on the bed in her husband's body. As a mother, you have the intuition to judge what kind of position most comfortable.

Day 259: Contractions may sometimes cause pain that even affect normal language dialogue, how to cope with contractions? Please take a deep breath. When you take a deep breath, there is more oxygen to be delivered to your uterus and the fetus, and lead to mild pain relief.

Day 266: Labor pain is not persistent pain, but a wave of pain. After each contraction, please relax to better cope with the next contraction.

Day 270: Moderate exercise can help start the delivery. Walking, climbing stairs and pre-gymnastics recommended sports. But you should have company during exercise.

All texts group
Includes all messages listed above from all groups. Messages sent to this group were sent on the same days as in the other groups.
