# Supplementary material for: Effect of a Text Messaging–Based Educational Intervention on Cesarean Section Rates Among Pregnant Women in China: Quasirandomized Controlled Trial
Source: JMIR Mhealth Uhealth. 2020 Nov 3;8(11):e19953. doi: 10.2196/19953 (PMC7671841; doi:10.2196/19953)
Supplement: Multimedia Appendix 3 [file mhealth_v8i11e19953_app3.pdf]

## Multimedia Appendix 3 Balance check, all baseline variables, all enrollees

| | Full sample | Basic only | Care seeking | House-hold practices | All texts | Test stat | P-value |
|---|---|---|---|---|---|---|---|
| **Age (yrs) λ** | 27.5 | 27.5 | 27.5 | 27.4 | 27.6 | F: 0.32 | 0.811 |
| SD | 3.9 | 4.0 | 3.9 | 3.8 | 3.9 | | |
| **Height (cm) λ** | 160.8 | 160.9 | 160.9 | 160.8 | 160.6 | F: 1.25 | 0.289 |
| SD | 4.7 | 4.8 | 4.8 | 4.7 | 4.4 | | |
| **Weight (Kg) λ** | 62.0 | 61.8 | 62.4 | 61.7 | 62.2 | F: 0.33 | 0.807 |
| SD | 20.0 | 19.2 | 20.2 | 20.3 | 20.3 | | |
| **Han (%)** | 99.1 | 99.4 | 99.1 | 99.3 | 98.7 | Chi2: 3.33 | 0.343 |
| Other Ethnicities | 0.9 | 0.6 | 0.9 | 0.7 | 1.3 | | |
| **Phone self-owned (%)** | 91.5 | 91.6 | 90.8 | 91.6 | 92.1 | Chi2: 1.49 | 0.684 |
| Use Others' Phone | 8.5 | 8.4 | 9.2 | 8.4 | 7.9 | | |
| **Currently married (%)** | 98.5 | 98.5 | 98.6 | 98.6 | 98.1 | Chi2: 1.31 | 0.726 |
| Other status | 1.5 | 1.5 | 1.4 | 1.4 | 1.9 | | |
| **Household members λ** | 4.3 | 4.3 | 4.2 | 4.3 | 4.3 | F: 0.48 | 0.695 |
| SD | 1.3 | 1.3 | 1.3 | 1.3 | 1.3 | | |
| **Monthly expenditures (RMB) - Mean** | 33,850 | 33,540 | 33,086 | 33,713 | 34,902 | F: 0.20 | 0.899 |
| SD | 43,242 | 44,939 | 38,918 | 34,684 | 51,360 | | |
| **Monthly income (RMB) λ** | 60,267 | 57,303 | 56,516 | 71,626 | 56,203 | F: 2.03 | 0.107 |
| SD | 128,128 | 73,563 | 72,770 | 231,390 | 56,501 | | |
| **a141** | 5.7 | 5.8 | 5.6 | 5.7 | 5.7 | F: 0.77 | 0.509 |
| Std. Dev. | 1.9 | 2.0 | 1.8 | 1.9 | 1.9 | | |
| **Eldest child's age ψ λ** | 6.0 | 5.9 | 6.0 | 5.9 | 6.1 | F: 0.43 | 0.730 |
| Std. Dev. | 3.6 | 3.6 | 3.6 | 3.6 | 3.5 | | |
| **Previous breastfeeding (mo) ψ λ** | 8.1 | 7.7 | 8.0 | 8.2 | 8.2 | F: 0.93 | 0.425 |
| Std. Dev. | 4.8 | 4.8 | 4.8 | 4.7 | 4.8 | | |
| **Gestational week at enrollment λ** | 15.1 | 14.9 | 15.2 | 15.2 | 15.1 | F: 0.38 | 0.764 |
| SD | 7.5 | 7.3 | 7.4 | 7.4 | 7.7 | | |
| **Residency (%)** | | | | | | Chi2: 11.36 | 0.252 |
| Province/City | 2.9 | 2.4 | 2.7 | 2.6 | 3.7 | | |
| County | 14.8 | 13.6 | 15.4 | 16.2 | 14.0 | | |
| Township | 18.5 | 20.7 | 17.9 | 17.7 | 17.8 | | |
| Village | 63.9 | 63.3 | 64.0 | 63.5 | 64.5 | | |
| **Occupation (%)** | | | | | | Chi2: 15.28 | 0.643 |
| Farmer | 20.4 | 21.5 | 19.7 | 21.2 | 19.4 | | |
| Business owner | 7.1 | 7.5 | 7.3 | 6.5 | 7.2 | | |
| Government worker | 4.1 | 3.7 | 4.3 | 4.7 | 3.9 | | |
| Migrant worker | 6.8 | 6.5 | 7.5 | 6.4 | 6.9 | | |
| Local worker | 5.0 | 3.7 | 4.9 | 6.1 | 5.2 | | |
| Home maker | 36.1 | 36.8 | 36.9 | 33.7 | 36.8 | | |
| Others | 20.4 | 20.4 | 19.4 | 21.4 | 20.7 | | |
| **Education level (%)** | | | | | | Chi2: 10.37 | 0.321 |
| Jr. High or less | 43.0 | 45.9 | 43.5 | 40.1 | 42.6 | | |
| Sr. High / Technical | 28.2 | 27.5 | 27.2 | 30.5 | 27.7 | | |
| 3 Yr. college | 21.1 | 19.7 | 21.6 | 21.9 | 21.3 | | |
| 4 Yr college + | 7.7 | 6.9 | 7.7 | 7.5 | 8.4 | | |
| **Husband education (%)** | | | | | | Chi2: 8.09 | 0.525 |
| Jr. High or less | 43.4 | 45.0 | 42.6 | 42.5 | 43.4 | | |
| Sr. High / technical | 28.5 | 29.0 | 30.0 | 27.3 | 27.9 | | |
| 3 Yr. college | 19.3 | 18.4 | 19.0 | 20.8 | 19.1 | | |
| 4 Yr college + | 8.8 | 7.7 | 8.4 | 9.4 | 9.7 | | |
| **Insurance (%)** | | | | | | Chi2: 9.45 | 0.664 |
| NCRMS | 77.4 | 78.9 | 77.0 | 76.3 | 77.5 | | |
| 2 | 6.8 | 5.4 | 7.4 | 7.2 | 7.0 | | |
| 3 | 8.7 | 9.5 | 8.5 | 8.5 | 8.3 | | |
| 4 | 2.1 | 2.1 | 2.2 | 2.3 | 1.7 | | |
| 5 | 5.0 | 4.1 | 4.9 | 5.7 | 5.5 | | |

| Multimedia appendix 3 continued | Full sample | Basic only | Care seeking | House-hold practices | All texts | Test stat | P-value |
|---|---|---|---|---|---|---|---|
| **Pregnancy Number (%)** | | | | | | Chi2: 1.49 | 0.960 |
| 1st | 43.2 | 42.9 | 43.7 | 43.5 | 42.7 | | |
| 2nd | 35.1 | 35.8 | 35.2 | 33.9 | 35.4 | | |
| 3rd + | 21.8 | 21.3 | 21.2 | 22.7 | 21.9 | | |
| Any past live births (%) | 35.6 | 36.0 | 34.7 | 35.7 | 36.0 | Chi2: 0.611 | 0.894 |
| No past live births (%) | 64.4 | 64.0 | 65.3 | 64.3 | 64.0 | | |
| Any past miscarriages (%) | 42.6 | 41.3 | 43.6 | 43.1 | 42.6 | Chi2: 1.30 | 0.729 |
| No past miscarriages (%) | 57.4 | 58.7 | 56.5 | 56.9 | 57.4 | | |
| **Previous delivery method (%) ψ** | | | | | | Chi2: 5.58 | 0.134 |
| Caesarean | 20.9 | 16.9 | 21.8 | 21.3 | 23.4 | | |
| Vaginal | 79.1 | 83.1 | 78.2 | 78.7 | 76.6 | | |
| **Previous delivery gender (%) ψ** | | | | | | Chi2: 0.51 | 0.917 |
| Female (%) | 63.4 | 64.1 | 62.6 | 64.4 | 62.5 | | |
| Male (%) | 36.7 | 35.9 | 37.4 | 35.6 | 37.5 | | |
| **Previous birth preterm (%) ψ** | | | | | | Chi2: 5.48 | 0.140 |
| Yes (%) | 5.1 | 7.2 | 3.7 | 5.3 | 4.3 | | |
| No (%) | 94.9 | 92.8 | 96.3 | 94.7 | 95.7 | | |
| **Health condition before Pregnancy (%)** | | | | | | Chi2: 8.96 | 0.441 |
| Very Good | 8.2 | 8.6 | 7.7 | 8.0 | 8.3 | | |
| Good | 49.6 | 47.1 | 50.1 | 50.1 | 50.7 | | |
| Fair | 40.6 | 42.6 | 40.4 | 40.9 | 38.9 | | |
| Poor / Very Poor | 1.6 | 1.7 | 1.8 | 0.9 | 2.1 | | |
| **Health compared to before pregnancy (%)** | | | | | | Chi2: 14.87 | 0.094* |
| Better | 4.4 | 4.0 | 4.6 | 5.3 | 3.8 | | |
| The same | 63.3 | 62.8 | 60.9 | 65.5 | 64.0 | | |
| Worse | 20.6 | 21.7 | 23.0 | 17.9 | 19.6 | | |
| Don't know | 11.8 | 11.6 | 11.5 | 11.3 | 12.6 | | |
| **Current smoker (%)** | | | | | | Chi2: 4.66 | 0.199 |
| Yes | 1.2 | 1.7 | 0.8 | 0.9 | 1.4 | | |
| No | 98.8 | 98.3 | 99.2 | 99.1 | 98.6 | | |
| **Husband smoke (%)** | | | | | | Chi2: 11.70 | 0.069* |
| Yes | 54.7 | 56.0 | 56.2 | 51.3 | 55.0 | | |
| No | 39.3 | 37.7 | 37.5 | 43.6 | 38.6 | | |
| Former | 6.1 | 6.3 | 6.4 | 5.1 | 6.3 | | |
| **Current drinker (%)** | | | | | | Chi2: 2.83 | 0.419 |
| Yes | 1.4 | 1.0 | 1.8 | 1.1 | 1.5 | | |
| No | 98.6 | 99.0 | 98.3 | 98.9 | 98.5 | | |
| **Husband Drink (%)** | | | | | | Chi2: 4.11 | 0.662 |
| Yes | 19.7 | 20.7 | 19.5 | 20.1 | 18.7 | | |
| No | 71.6 | 70.2 | 72.4 | 71.8 | 71.7 | | |
| Former | 8.7 | 9.1 | 8.1 | 8.1 | 9.6 | | |
| **Exerciser (%)** | | | | | | Chi2: 3.37 | 0.762 |
| Yes | 33.6 | 34.7 | 33.1 | 34.3 | 32.4 | | |
| No | 55.5 | 55.4 | 55.5 | 54.3 | 56.5 | | |
| Former | 11.0 | 9.9 | 11.4 | 11.5 | 11.1 | | |
| **Husband exerciser (%)** | | | | | | Chi2: 2.48 | 0.871 |
| Yes | 39.9 | 38.9 | 39.8 | 41.2 | 39.8 | | |
| No | 53.6 | 54.2 | 53.5 | 53.2 | 53.6 | | |
| Former | 6.5 | 6.9 | 6.7 | 5.6 | 6.7 | | |
| **Health institution (%)** | | | | | | Chi2: 3.24 | 0.356 |
| 1 | 12.2 | 11.3 | 11.9 | 11.8 | 13.6 | | |
| 2 | 87.8 | 88.7 | 88.1 | 88.2 | 86.4 | | |
| **Internet (%)** | | | | | | Chi2: 3.48 | 0.324 |
| 1 | 43.9 | 43.5 | 43.1 | 42.7 | 46.2 | | |
| 2 | 56.1 | 56.5 | 56.9 | 57.3 | 53.8 | | |

| Multimedia appendix 3 continued | Full sample | Basic only | Care seeking | House-hold practices | All texts | Test stat | P-value |
|---|---|---|---|---|---|---|---|
| **Television (%)** | | | | | | | |
| 1 | 8.3 | 8.4 | 8.2 | 8.0 | 8.7 | Chi2: 0.37 | 0.946 |
| 2 | 91.7 | 91.6 | 91.8 | 92.0 | 91.4 | | |
| **Books (%)** | | | | | | | |
| 1 | 25.3 | 25.1 | 24.9 | 27.6 | 23.9 | Chi2: 4.12 | 0.249 |
| 2 | 74.7 | 74.9 | 75.1 | 72.4 | 76.1 | | |
| **Friends (%)** | | | | | | | |
| 1 | 33.7 | 33.3 | 35.2 | 33.9 | 32.4 | Chi2: 1.96 | 0.581 |
| 2 | 66.3 | 66.7 | 64.9 | 66.1 | 67.6 | | |
| **Family (%)** | | | | | | | |
| 1 | 14.7 | 15.4 | 13.3 | 15.0 | 15.2 | Chi2: 2.31 | 0.510 |
| 2 | 85.3 | 84.6 | 86.7 | 85.0 | 84.8 | | |
| **None (%)** | | | | | | | |
| 1 | 4.6 | 4.9 | 3.8 | 4.6 | 5.0 | Chi2: 2.04 | 0.564 |
| 2 | 95.4 | 95.1 | 96.2 | 95.4 | 95.1 | | |
| **Others (%)** | | | | | | | |
| 1 | 1.8 | 2.4 | 1.7 | 1.8 | 1.4 | Chi2: 2.84 | 0.417 |
| 2 | 98.2 | 97.7 | 98.3 | 98.2 | 98.6 | | |
| **Planned pregnancy (%)** | | | | | | | |
| Yes | 65.9 | 64.2 | 66.7 | 67.0 | 65.9 | Chi2: 2.25 | 0.523 |
| No | 34.1 | 35.8 | 33.3 | 33.1 | 34.1 | | |
| **a24** | | | | | | | |
| Singleton | 85.9 | 85.5 | 86.1 | 86.3 | 85.5 | Chi2: 1.79 | 0.938 |
| Twins + | 1.0 | 0.8 | 1.1 | 1.2 | 1.1 | | |
| Unsure | 13.1 | 13.7 | 12.8 | 12.5 | 13.4 | | |
| **Attitudes (%)** | | | | | | | |
| 1 | 5.8 | 6.1 | 5.7 | 6.5 | 5.0 | | |
| 2 | 0.4 | 0.4 | 0.5 | 0.4 | 0.5 | | |
| 3 | 17.8 | 18.2 | 17.5 | 17.7 | 17.7 | Chi2: 10.51 | 0.787 |
| 4 | 50.1 | 50.4 | 50.3 | 47.8 | 51.8 | | |
| 5 | 24.6 | 23.7 | 24.3 | 26.2 | 24.2 | | |
| Don't Know | 1.3 | 1.2 | 1.7 | 1.4 | 0.8 | | |
| **Expectations (%)** | | | | | | | |
| 1 | 1.3 | 1.4 | 1.4 | 1.3 | 1.3 | | |
| 2 | 1.0 | 0.7 | 1.0 | 1.0 | 1.3 | | |
| 3 | 28.9 | 29.0 | 28.0 | 29.8 | 28.8 | Chi2: 8.03 | 0.923 |
| 4 | 52.1 | 53.1 | 51.5 | 52.6 | 51.3 | | |
| 5 | 12.0 | 11.2 | 12.8 | 11.3 | 12.4 | | |
| Don't Know | 4.8 | 4.6 | 5.4 | 3.9 | 5.0 | | |
| **Self-efficacy (%)** | | | | | | | |
| 1 | 1.1 | 1.6 | 1.1 | 1.0 | 0.8 | | |
| 2 | 20.2 | 19.1 | 19.7 | 20.4 | 21.4 | | |
| 3 | 47.2 | 48.6 | 47.1 | 47.7 | 45.5 | Chi2: 10.61 | 0.780 |
| 4 | 16.6 | 15.9 | 17.1 | 16.4 | 17.0 | | |
| 5 | 9.5 | 10.1 | 9.3 | 9.6 | 9.2 | | |
| Don't know | 5.4 | 4.8 | 5.7 | 4.8 | 6.1 | | |
| **Personal norms (%)** | | | | | | | |
| 1 | 24.3 | 25.5 | 25.2 | 22.4 | 23.9 | | |
| 2 | 34.9 | 35.8 | 33.3 | 35.8 | 35.0 | | |
| 3 | 24.7 | 21.9 | 24.3 | 27.0 | 25.7 | Chi2: 21.20 | 0.131 |
| 4 | 10.0 | 10.2 | 10.3 | 9.8 | 9.7 | | |
| 5 | 3.1 | 4.0 | 2.8 | 2.5 | 3.0 | | |
| Don't know | 3.0 | 2.6 | 4.1 | 2.6 | 2.8 | | |
| **Intentions (%)** | | | | | | | |
| 1 | 1.7 | 1.3 | 3.0 | 1.5 | 1.1 | Chi2: 25.68 | 0.042** |
| 2 | 4.9 | 4.3 | 5.2 | 4.8 | 5.2 | | |
| 3 | 36.5 | 36.9 | 36.6 | 36.2 | 36.4 | | |

| | | | | | | | |
|---|---|---|---|---|---|---|---|
| 4 | 38.5 | 38.1 | 37.2 | 40.8 | 38.0 | | |
| 5 | 15.8 | 16.4 | 15.4 | 15.1 | 16.1 | | |
| Don't know | 2.6 | 3.0 | 2.6 | 1.6 | 3.3 | | |
| **Multimedia appendix 3 continued** | **Full sample** | **Basic only** | **Care seeking** | **House-hold practices** | **All texts** | **Test stat** | **P-value** |
| **Plans (%)** | | | | | | | |
| 1 | 13.2 | 13.0 | 14.0 | 13.1 | 12.8 | | |
| 2 | 32.1 | 30.6 | 33.9 | 30.7 | 33.1 | | |
| 3 | 40.9 | 43.0 | 37.7 | 42.8 | 40.3 | Chi2: 15.96 | 0.385 |
| 4 | 7.2 | 6.2 | 7.7 | 7.5 | 7.3 | | |
| 5 | 3.5 | 4.1 | 3.0 | 3.2 | 3.8 | | |
| Don't Know | 3.1 | 3.2 | 3.7 | 2.7 | 2.7 | | |
| **Susceptibility (%)** | | | | | | | |
| 1 | 23.1 | 22.9 | 22.4 | 23.2 | 23.9 | | |
| 2 | 18.0 | 17.3 | 19.4 | 17.8 | 17.5 | | |
| 3 | 16.2 | 16.1 | 15.3 | 17.4 | 15.9 | Chi2: 9.47 | 0.852 |
| 4 | 4.7 | 5.3 | 4.7 | 4.5 | 4.4 | | |
| 5 | 14.1 | 15.1 | 12.6 | 14.3 | 14.4 | | |
| Don't Know | 23.9 | 23.3 | 25.5 | 22.7 | 23.9 | | |
| **Severity (%)** | | | | | | | |
| 1 | 18.2 | 18.1 | 17.5 | 19.5 | 18.0 | | |
| 2 | 19.0 | 18.7 | 19.8 | 18.8 | 18.7 | | |
| 3 | 7.6 | 7.4 | 6.6 | 9.3 | 7.0 | Chi2: 11.76 | 0.697 |
| 4 | 3.4 | 3.1 | 4.1 | 3.3 | 3.1 | | |
| 5 | 15.2 | 15.1 | 15.3 | 14.7 | 15.5 | | |
| Don't Know | 36.7 | 37.6 | 36.8 | 34.5 | 37.7 | | |
| **Social norms (%)** | | | | | | | |
| 1 | 1.1 | 0.9 | 1.4 | 1.1 | 1.1 | | |
| 2 | 7.7 | 8.8 | 8.4 | 6.6 | 6.9 | | |
| 3 | 10.3 | 10.8 | 9.9 | 9.3 | 11.0 | Chi2: 12.36 | 0.652 |
| 4 | 55.9 | 55.2 | 56.1 | 57.9 | 54.6 | | |
| 5 | 7.6 | 7.6 | 6.7 | 8.0 | 8.2 | | |
| Don't Know | 17.5 | 16.9 | 17.6 | 17.1 | 18.2 | | |
| **Family preference for gender (%)** | | | | | | | |
| Boy | 8.0 | 8.3 | 7.0 | 7.9 | 8.8 | Chi2: 5.77 | 0.499 |
| Girl | 7.7 | 7.6 | 7.7 | 6.6 | 8.5 | | |
| No preference | 84.4 | 84.2 | 85.3 | 85.5 | 82.7 | | |
| **Self-preference for gender (%)** | | | | | | | |
| Boy | 7.6 | 9.1 | 6.3 | 7.1 | 7.9 | Chi2: 10.90 | 0.092 |
| Girl | 19.8 | 21.3 | 19.3 | 20.3 | 18.4 | | |
| No preference | 72.7 | 69.6 | 74.4 | 72.6 | 73.7 | | |
| **Preference For Delivery (%)** | | | | | | | |
| Vaginal | 83.5 | 84.9 | 85.2 | 82.3 | 81.8 | Chi2: 9.49 | 0.148 |
| Caesarean | 6.7 | 6.0 | 6.4 | 7.6 | 6.8 | | |
| Don't know | 9.8 | 9.2 | 8.4 | 10.1 | 11.4 | | |
| **Reason prefer caesarean (%) θ** | | | | | | | |
| Vaginal is painful | 20.3 | 24.1 | 22.0 | 16.8 | 18.7 | | |
| My friends choose it | 7.7 | 3.6 | 6.0 | 14.9 | 5.5 | Chi2: 12.24 | 0.200 |
| Doctors suggested | 52.0 | 54.2 | 54.0 | 47.5 | 52.8 | | |
| Other | 20.0 | 18.1 | 18.0 | 20.8 | 23.1 | | |

λ = Row represents mean values in each group

ψ = Asked only if respondent had previous children; % denote rates amongst this subset of women.

θ = Asked only if stated preferred a caesarean delivery; % denote rates amongst this subset of women.

* p < .10

** p < .05
