# Supplementary material for: Effect of a Text Messaging–Based Educational Intervention on Cesarean Section Rates Among Pregnant Women in China: Quasirandomized Controlled Trial
Source: JMIR Mhealth Uhealth. 2020 Nov 3;8(11):e19953. doi: 10.2196/19953 (PMC7671841; doi:10.2196/19953)
Supplement: Multimedia Appendix 4 [file mhealth_v8i11e19953_app4.pdf]

Multimedia Appendix 4 Balance check, all baseline variables, women with follow-up surveys

	Full sample	Basic only	Care seeking	House-hold practices	All texts	Test stat	P-value
**Age (yrs) λ**	27.4	27.3	27.5	27.4	27.4	F: 0.31	0.818
SD	3.9	3.8	4.0	3.9	3.8		
**Height (cm) λ**	160.8	161.0	160.8	160.8	160.6	F: 0.70	0.550
SD	4.8	5.1	4.7	4.7	4.5		
**Weight (Kg) λ**	62.1	62.3	62.8	61.8	61.4	F: 0.50	0.683
SD	19.9	19.2	21.0	20.2	19.4		
**Han (%)**	99.3	99.8	99.4	99.4	99.1	Chi2: 2.26	0.521
Other ethnicities	0.7	0.2	0.6	0.6	0.9		
**Phone self-owned (%)**	99.0	93.0	91.1	91.2	92.4	Chi2: 1.91	0.592
Use others' phone	1.0	7.0	8.9	8.8	7.6		
**Currently married (%)**	99.0	98.8	98.6	99.2	99.3	Chi2: 1.51	0.679
Other status	1.0	1.2	1.4	0.8	0.7		
**Household members λ**	4.4	4.4	4.4	4.3	4.4	F: 1.04	0.374
SD	1.2	1.2	1.2	1.2	1.2		
**Monthly expenditures (RMB) - Mean**	33,600	33,164	30,275	33,958	36,284	F: 0.70	0.554
SD	47,317	55,023	35,259	40,371	53,163		
**Monthly income (RMB) λ**	59,444	49,747	57,278	75,245	55,863	F: 1.19	0.312
SD	162,790	47,551	86,424	310,236	52,945		
**a141**	5.66	5.81	5.62	5.64	5.57	F: 1.04	0.375
Std. Dev.	1.92	1.87	1.86	1.96	1.99		
**Eldest child's age ψ λ**	6.0	5.7	5.9	6.0	6.1	F: 0.47	0.705
Std. Dev.	3.5	3.5	3.6	3.6	3.4		
**Previous breastfeeding (mo) ψ λ**	8.1	7.9	8.0	8.3	8.3	F: 0.27	0.849
Std. Dev.	4.8	4.6	4.8	5.0	4.9		
**Gestational week at enrollment λ**	14.9	14.3	15.6	14.9	14.7	F: 2.43	0.064*
SD	7.4	7.2	7.7	7.4	7.2		
**Residency (%)**						Chi2: 13.09	0.162
Province/City	2.4	2.0	2.4	1.7	3.6		
County	8.8	8.2	8.7	8.7	9.6		
Township	17.5	21.0	18.3	16.2	14.6		
Village	71.3	68.8	70.6	73.4	72.2		
**Occupation (%)**						Chi2: 11.94	0.851
Farmer	22.4	23.5	21.4	24.7	20.5		
Buisness owner	6.1	6.3	6.2	5.9	5.9		
Government worker	2.2	2.6	2.8	1.9	1.8		
Migrant worker	5.5	5.1	6.8	4.6	5.5		
Local worker	4.0	3.3	3.4	4.2	4.8		
Home maker	38.7	38.2	40.2	38.5	37.9		
Others	21.1	21.0	19.2	20.3	23.7		
**Education Level (%)**						Chi2: 5.51	0.788
Jr. High or less	45.0	48.2	46.2	43.7	42.3		
Sr. High / Technical	30.1	29.3	29.3	31.2	30.6		
3 Yr. college	19.3	17.2	19.1	19.9	21.1		
4 Yr college +	5.6	5.4	5.5	5.3	6.0		
**Husband education (%)**						Chi2: 8.64	0.471
Jr. High or less	44.7	43.4	45.6	46.8	43.4		
Sr. High / Technical	31.4	34.9	31.4	27.2	31.8		
3 Yr. college	16.8	14.9	16.1	18.2	18.0		
4 Yr college +	7.1	6.8	6.9	7.8	6.9		
**Insurance (%)**						Chi2: 13.06	0.365
NCRMS	82.0	82.7	81.5	80.9	82.9		
2	3.5	2.5	4.4	3.6	3.4		
3	8.2	8.8	8.6	7.9	7.5		
4	2.1	2.5	2.7	1.9	1.1		
5	4.3	3.6	2.7	5.6	5.1		

Multimedia Appendix 4 Continued	Full sample	Basic only	Care seeking	House-hold practices	All texts	Test stat	P-value
**Pregnancy number (%)**							
1st	42.8	44.1	42.0	43.4	41.7	Chi2: 5.03	0.539
2nd	35.0	36.6	35.4	32.4	35.3		
3rd +	22.3	19.3	22.7	24.2	23.0		
Any past live births (%)	36.7	35.8	37.8	35.3	37.5	Chi2: 0.948	0.814
No past live births (%)	63.4	64.2	62.2	64.7	62.5		
Any past miscarriages (%)	42.7	37.7	44.9	44.7	43.7	Chi2: 7.28	0.063*
No past miscarriages (%)	57.3	62.4	55.2	55.3	56.3		
**Previous delivery method (%) ψ**							
Caesarean	15.4	21.7	18.3	20.2	19.0	Chi2: 2.68	0.444
Vaginal	84.6	78.4	81.7	79.8	81.0		
**Previous delivery gender (%) ψ**							
Female (%)	63.4	62.6	62.8	65.3	63.1	Chi2: 0.357	0.949
Male (%)	36.6	37.4	37.2	34.7	36.9		
**Previous birth preterm (%) ψ**							
Yes (%)	4.4	6.0	4.8	4.2	3.0	Chi2: 2.1	0.553
No (%)	95.6	94.0	95.2	95.8	97.0		
**Health condition before pregnancy (%)**							
Very good	8.4	8.4	7.6	8.8	8.8	Chi2: 2.19	0.988
Good	49.6	48.5	49.5	50.7	49.7		
Fair	40.3	41.7	41.2	39.0	39.5		
Poor / Very poor	1.7	1.4	1.8	1.5	2.0		
**Health compared to before pregnancy (%)**							
Better	4.4	3.6	4.6	5.1	4.2	Chi2: 4.93	0.840
The same	64.1	63.4	62.3	66.3	64.6		
Worse	20.5	22.0	22.2	18.1	19.7		
Don't know	11.0	10.9	10.9	10.5	11.6		
**Current smoker (%)**							
Yes	1.1	2.2	1.0	0.4	0.9	Chi2: 7.42	0.060*
No	98.9	97.9	99.0	99.6	99.1		
**Husband smoke (%)**							
Yes	56.9	57.7	59.4	52.7	57.6	Chi2: 8.75	0.188
No	37.2	35.6	35.4	42.3	35.8		
Former	5.9	6.7	5.2	5.0	6.6		
**Current drinker (%)**							
Yes	1.5	0.6	2.4	1.3	1.8	Chi2: 6.09	0.107
No	98.5	99.4	97.6	98.8	98.2		
**Husband drink (%)**							
Yes	100.0	26.2	24.3	23.8	25.7	Chi2: 7.18	0.304
No	100.0	24.2	25.3	23.5	27.1		
Former	100.0	27.9	17.9	21.8	32.4		
**Exerciser (%)**							
Yes	33.4	34.8	33.3	33.3	32.3	Chi2: 1.77	0.939
No	55.6	55.5	55.6	55.4	55.9		
Former	11.0	9.7	11.0	11.3	11.8		
**Husband exerciser (%)**							
Yes	38.6	39.3	37.5	39.1	38.7	Chi2: 7.65	0.265
No	55.0	53.3	57.1	56.5	53.5		
Former	6.4	7.5	5.4	4.4	7.8		
**Health institution (%)**							
1	11.6	9.8	12.0	11.3	13.4	Chi2: 3.37	0.338
2	88.4	90.2	88.0	88.7	86.6		
**Internet (%)**							
1	43.3	43.1	42.2	43.6	44.1	Chi2: 0.39	0.943
2	56.7	56.9	57.8	56.4	55.9		
**Multimedia Appendix 4 Continued**	**Full**	**Basic**	**Care**	**House-hold**	**All**	**Test stat**	**P-value**

	sample	only	seeking	practices	texts		
**Television (%)**							
1	8.2	7.9	8.0	9.3	7.5	Chi2: 1.19	0.755
2	91.8	92.1	92.0	90.7	92.5		
**Books (%)**							
1	25.8	25.8	26.2	26.4	24.9	Chi2: 0.351	0.950
2	74.2	74.2	73.8	73.6	75.1		
**Friends (%)**							
1	34.5	34.4	35.0	35.9	32.8	Chi2: 1.18	0.757
2	65.5	65.6	65.0	64.1	67.2		
**Family (%)**							
1	14.2	15.1	13.0	14.5	14.2	Chi2: 0.888	0.828
2	85.8	84.9	87.0	85.5	85.9		
**None (%)**							
1	4.9	4.9	4.7	4.8	5.1	Chi2: 0.126	0.988
2	95.1	95.1	95.4	95.2	94.9		
**Others (%)**							
1	1.8	2.5	1.3	1.3	2.1	Chi2: 2.79	0.426
2	98.2	97.5	98.7	98.7	97.9		
**Planned pregnancy (%)**							
Yes	65.8	63.4	66.7	68.7	64.9	Chi2: 3.51	0.319
No	34.2	36.7	33.3	31.3	35.1		
**a24**							
Singleton	85.2	84.7	85.5	85.2	85.5	Chi2: 1.28	0.973
Twins +	0.9	0.6	0.8	1.1	1.1		
Unsure	13.9	14.7	13.6	13.7	13.4		
**Attitudes (%)**							
1	4.7	6.1	4.8	4.0	3.9		
2	0.6	0.6	1.0	0.2	0.7		
3	17.2	20.0	16.1	16.0	16.6	Chi2: 21.51	0.121
4	51.9	51.1	52.9	49.9	53.4		
5	24.5	21.3	23.7	27.9	25.1		
Don't know	1.2	1.0	1.6	2.1	0.4		
**Expectations (%)**							
1	1.1	1.6	1.0	1.3	0.5		
2	0.9	0.2	1.4	0.4	1.4		
3	28.3	30.1	27.0	28.2	27.8	Chi2: 16.15	0.372
4	52.8	52.1	52.4	53.9	53.0		
5	11.8	9.8	12.8	12.0	12.6		
Don't know	5.1	6.3	5.4	4.2	4.7		
**Self-efficacy (%)**							
1	1.1	1.6	1.4	1.1	0.4		
2	20.6	18.9	21.8	20.4	21.3		
3	47.0	51.4	44.6	46.6	45.6	Chi2: 12.31	0.655
4	16.9	15.7	16.9	17.0	17.9		
5	9.0	8.3	9.2	9.9	8.9		
Don't know	5.3	4.2	6.1	5.0	6.0		
**Personal norms (%)**							
1	25.0	26.4	26.1	21.7	25.5		
2	34.8	34.1	32.9	37.4	34.8		
3	24.2	22.6	24.9	25.7	23.9	Chi2: 13.92	0.532
4	9.8	10.0	9.8	9.4	9.8		
5	3.0	4.5	2.4	2.1	2.9		
Don't know	3.3	2.4	4.0	3.8	3.0		
**Intentions (%)**							
1	2.2	1.6	4.3	1.5	1.6		
2	4.9	4.7	4.3	5.2	5.4	Chi2: 26.12	0.037**
3	35.1	39.5	35.2	32.6	33.1		
4	39.3	35.5	37.0	43.7	41.1		

5	16.0	15.8	17.4	14.2	16.6		
Don't know	2.4	3.0	1.8	2.7	2.2		
**Multimedia Appendix 4 Continued**	**Full sample**	**Basic only**	**Care seeking**	**House-hold practices**	**All texts**	**Test stat**	**P-value**
**Plans (%)**						Chi2: 8.26	0.913
1	12.2	12.4	12.1	11.7	12.4		
2	33.2	33.3	32.8	33.7	33.0		
3	40.4	42.4	40.2	40.2	39.0		
4	7.7	5.9	7.9	7.5	9.2		
5	3.4	2.6	3.4	3.6	4.1		
Don't know	3.1	3.4	3.6	3.4	2.3		
**Susceptibility (%)**						Chi2: 15.57	0.411
1	24.3	23.5	23.3	23.6	26.6		
2	17.1	14.7	18.4	17.4	17.8		
3	15.4	15.3	13.6	17.0	15.8		
4	5.1	6.5	4.8	4.4	4.9		
5	13.7	16.1	12.2	13.0	13.3		
Don't know	24.4	23.9	27.7	24.7	21.6		
**Severity (%)**						Chi2: 8.03	0.923
1	20.1	19.2	19.5	20.9	20.8		
2	17.6	16.7	18.3	18.5	16.9		
3	7.9	8.6	6.2	9.1	7.8		
4	2.8	3.3	2.7	2.6	2.6		
5	14.1	15.9	13.5	13.9	13.2		
Don't know	37.5	36.3	39.7	35.0	38.8		
**Social norms (%)**						Chi2: 19.66	0.185
1	1.2	1.4	1.6	0.9	0.9		
2	8.6	10.7	10.3	6.0	7.6		
3	10.4	10.3	10.3	8.7	12.1		
4	54.6	51.8	53.8	60.4	52.9		
5	7.8	7.3	8.3	7.0	8.6		
Don't know	17.4	18.6	15.7	17.0	18.0		
**Family preference for gender (%)**						Chi2: 2.2	0.900
Boy	7.9	7.7	7.2	7.8	8.7		
Girl	8.2	9.1	8.2	7.1	8.4		
No preference	83.9	83.2	84.6	85.1	82.9		
**Self-preference for gender (%)**						Chi2: 10.32	0.112
Boy	7.5	8.1	6.4	7.1	8.1		
Girl	19.4	23.0	17.3	20.8	17.0		
No preference	73.1	68.9	76.3	72.1	75.0		
**Preference for delivery (%)**						Chi2: 4.18	0.652
Vaginal	84.0	83.2	84.5	84.9	83.3		
Caesarean	6.4	5.7	7.4	6.1	6.5		
Don't know	9.6	11.1	8.0	9.0	10.2		
**Reason prefer caesarean (%) θ**						Chi2: 10.1	0.343
Vaginal is painful	19.5	25.0	17.7	17.1	18.4		
My friends choose it	7.9	0.0	9.8	14.3	7.9		
Doctors suggested	53.1	50.0	60.8	51.4	47.4		
Other	19.5	25.0	11.8	17.1	26.3		

λ = Row represents mean values in each group
ψ = Asked only if respondent had previous children; % denote rates amongst this subset of women.
θ = Asked only if stated preferred a caesarean delivery; % denote rates amongst this subset of women.
* $p < .10$
** $p < .05$
