# Supplementary material for: Effect of a Text Messaging–Based Educational Intervention on Cesarean Section Rates Among Pregnant Women in China: Quasirandomized Controlled Trial
Source: JMIR Mhealth Uhealth. 2020 Nov 3;8(11):e19953. doi: 10.2196/19953 (PMC7671841; doi:10.2196/19953)
Supplement: Multimedia Appendix 5 [file mhealth_v8i11e19953_app5.pdf]

Multimedia Appendix 5 Reasons for current CS delivery by SMS intervention assignment (N=2,104)

Reason given for delivering via CS	Basic	Care seeking	Home practices	All texts
NA: Completed vaginal delivery	77.2%	82.0%	80.2%	83.9%
"Vaginal delivery failed"	4.6%	3.5%	3.9%	4.2%
"Due to previous caesarean"	0.4%	0.2%	0.2%	0.2%
Reason clearly medically indicated CS	6.5%	4.4%	3.8%	3.9%
Reason could indicate CS in some cases	2.2%	1.3%	2.1%	1.1%
"Doctor suggested"	4.5%	5.1%	6.1%	2.9%
"Other", not specified	3.8%	2.8%	2.3%	2.6%
Reply not a medical indication for CS	0.8%	0.8%	1.3%	1.1%
**Total**	100.0%	100.0%	100.0%	100.0%
