# Supplementary material for: Effect of a Text Messaging–Based Educational Intervention on Cesarean Section Rates Among Pregnant Women in China: Quasirandomized Controlled Trial
Source: JMIR Mhealth Uhealth. 2020 Nov 3;8(11):e19953. doi: 10.2196/19953 (PMC7671841; doi:10.2196/19953)
Supplement: Multimedia Appendix 6 [file mhealth_v8i11e19953_app6.pdf]

Multimedia Appendix 6 Differences in reasons for current CS delivery by SMS intervention assignment

Reason given for delivering via CS	Basic	Care seeking	Home practices	All texts
NA: Completed vaginal delivery	--	4.7%	3.0%	6.7%
"Vaginal delivery failed"	--	-1.1%	-0.6%	-0.4%
"Due to previous caesarean"	--	-0.2%	-0.2%	-0.2%
Reason clearly medically indicated CS	--	-2.0%	-2.6%	-2.5%
Reason could indicate CS in some cases	--	-1.0%	-0.2%	-1.1%
"Doctor suggested"	--	0.6%	1.6%	-1.6%
"Other", not specified	--	-1.1%	-1.5%	-1.2%
Reply not a medical indication for CS	--	0.0%	0.6%	0.3%
**Total**	--	0.0%	0.0%	0.0%
